# Purinergic Signaling in Controlling Macrophage and T Cell Functions During Atherosclerosis Development

**DOI:** 10.3389/fimmu.2020.617804

**Published:** 2021-02-16

**Authors:** Davide Ferrari, Andrea la Sala, Daniela Milani, Claudio Celeghini, Fabio Casciano

**Affiliations:** ^1^Department of Life Science and Biotechnology, Section of Microbiology and Applied Pathology, University of Ferrara, Ferrara, Italy; ^2^Certification Unit, Health Directorate, Bambino Gesù Pediatric Hospital, IRCCS, Rome, Italy; ^3^Department of Translational Medicine and LTTA Centre, University of Ferrara, Ferrara, Italy

**Keywords:** T lymphocytes, extracellular ATP and adenosine, CD39 and CD73, P1 and P2 receptors, atherosclerosis, macrophage, necrotic core, oxLDL

## Abstract

Atherosclerosis is a hardening and narrowing of arteries causing a reduction of blood flow. It is a leading cause of death in industrialized countries as it causes heart attacks, strokes, and peripheral vascular disease. Pathogenesis of the atherosclerotic lesion (atheroma) relies on the accumulation of cholesterol-containing low-density lipoproteins (LDL) and on changes of artery endothelium that becomes adhesive for monocytes and lymphocytes. Immunomediated inflammatory response stimulated by lipoprotein oxidation, cytokine secretion and release of pro-inflammatory mediators, worsens the pathological context by amplifying tissue damage to the arterial lining and increasing flow-limiting stenosis. Formation of thrombi upon rupture of the endothelium and the fibrous cup may also occur, triggering thrombosis often threatening the patient’s life. Purinergic signaling, i.e., cell responses induced by stimulation of P2 and P1 membrane receptors for the extracellular nucleotides (ATP, ADP, UTP, and UDP) and nucleosides (adenosine), has been implicated in modulating the immunological response in atherosclerotic cardiovascular disease. In this review we will describe advancements in the understanding of purinergic modulation of the two main immune cells involved in atherogenesis, i.e., monocytes/macrophages and T lymphocytes, highlighting modulation of pro- and anti-atherosclerotic mediated responses of purinergic signaling in these cells and providing new insights to point out their potential clinical significance.

## Introduction

**Figure 1:**
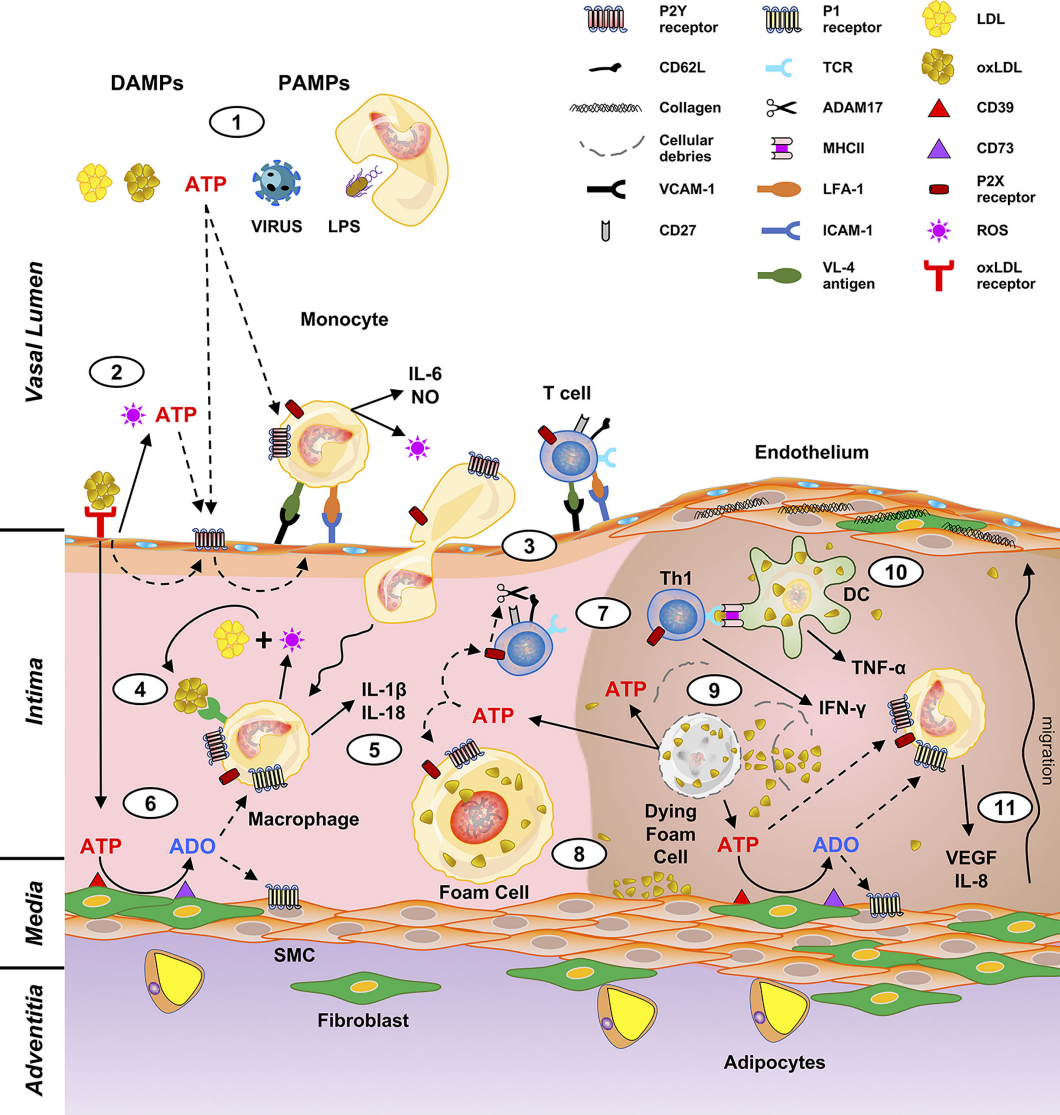
Putative role of purinergic signaling in driving macrophages and T cell activation in atheroma development. DAMPs (LDL, oxLDL, extracellular ATP) and PAMPs (viruses, microbes, LPS) trigger the production of cytokines and oxygen species by monocytes (1). This induces release of ATP from the endothelial cells and expression of the leukocyte adhesion molecules (vascular cell adhesion molecule 1, VCAM-1; intercellular adhesion molecule 1, ICAM-1) (2) thus prompting adhesion and extravasation of monocytes and lymphocytes (3). Macrophage derived ROS oxidate LDL to oxLDL (4) and stimulate IL-1β and IL-18 production (5). Extracellular ATP is also converted to ADO by CD39 and CD73, which are expressed by the intima cells (6). ADO exerts a down-modulation of the immune response therefore has a protective effect. On the contrary, ATP acts as a proinflammatory molecule inducing the cleavage of CD62L by ADAM17 and T cell polarization to a Th1 phenotype (7). Upon engulfment of oxLDL, macrophages become foam cells (8). The atheroma “necrotic core” (right part of the figure) forms by accumulation of dying foam cells, lipids, cholesterol crystals and immune cells (9). Pro-inflammatory IFN-γ and TNF-α are released upon antigen presentation to T lymphocytes by DC (10), and in turn promote IL-8 and VEGF secretion, with consequent fibroblasts and SMC migration and proliferation (11).

Atherosclerosis is a chronic inflammatory disease of the arteries, characterized by the development of characteristic lesions named atheromatous plaques (1, 2). It represents the most diffuse pathological state of peripheral and coronary artery disease, as well as of cerebrovascular disorders (3). Factors participating in the atherosclerotic process have been identified, among them: genetic predisposition, hyperlipidemia, metabolic dysregulation (obesity, diabetes), hypertension and smoking (4). A role for microorganisms has also been hypothesized, at least for the initial stages of atherosclerosis (**Figure 1**, topic 1) (5–8). The first steps of atherogenesis are characterized by endothelium activation and changes in lipid permeability. Expression of VCAM-1, ICAM-1, P-Selectin and different cytokine receptors allows endothelial adhesion of immune cells (monocytes, lymphocytes, neutrophils) (9). Permeation of cholesterol-containing low-density lipoproteins (LDL) in the inner lining of the artery wall and their oxidation (oxLDL) by reactive oxygen species (ROS) favor leukocyte activation and amplification of the pro-inflammatory background (**Figure 1**, topic 2) (1). Upon expression of scavenger receptors, engulfment of oxLDL and migration to the intima, circulating monocytes become macrophages (foam cells) that dying in the plaque release engulfed lipids (**Figure 1**, topics 3, 4). Necrotic immune cells, debris, extracellular lipids and cholesterol crystals are not cleared efficiently and accumulate within the plaque-forming the so-called “necrotic core” (**Figure 1**, topics 5, 8–9) (2).

Macrophages are central in atherosclerosis as they participate in all stages of atheroma formation (10–12). Circulating monocytes are captured by the activated endothelium and undergo differentiation into macrophages and changing their phenotype according to stages of the atherosclerotic process. They perform different tasks ranging from perception of danger signals, engulfment of lipids and dead cells, secretion of inflammatory (ROS, activating cytokines) but also pro-resolving molecules (12). Atheromas are mainly populated by pro-inflammatory M1 macrophages but also by DC able to perform antigen presentation to T lymphocytes (13, 14). Interestingly, while M1 macrophages promote atherogenesis, M2 are atheroprotective (15). Macrophages are activated by the Th1 cytokine IFN-γ to produce ROI and NO (**Figure 1**). IFN-γ is fundamental for the pathogenesis of atherosclerosis and endowed with the ability to cause atheroma even in the absence of immune cells (16, 17). Adaptive immunity takes part in the pathogenesis of atherosclerosis (18, 19). Although monocytes migrating through the intima are more abundant than T lymphocytes, these latter cells are crucial for the formation of the lesion as they produce activation signals for macrophages thus amplifying their contribution to atheroma formation (20). Differentiation of naïve CD4^+^ lymphocytes to effector and memory T cell subsets take place during atherogenesis (21). Antigen presentation by lesional macrophages and DC enables T cells to recognize antigens promoting the pro-inflammatory response underlying atherosclerosis (**Figure 1**, topic 10). Among them: LDL, oxLDL, beta 2 microglobulins, HSP60, and apo B-100 (22, 23). T cell polarization into Th1 and Th17 populations induce production of TNF-α, IL-17a and IFN-γ pro-inflammatory cytokines (16, 24, 25); however, Treg anti-inflammatory IL-10 and TGF-β cytokines have also been detected in the atheromatous lesions (23). Therefore, T cells secrete pro- and anti-inflammatory cytokines that direct evolution and stability of the plaque (19, 26).

Fibroblasts proliferate and secrete collagen, proteoglycans and elastin that accumulate in the intima (**Figure 1**, topic 11) (27). Immune cells promote not only atheroma formation but also its evolution with complications, damage, and sometimes disruption (23, 28). Thrombotic complications may also occur as a consequence of endothelial damage, rupture of the fibrous cap and exposure of prothrombotic material which triggers platelet activation and lead to blood coagulation. Plaque fracture is a very dangerous event threatening patients’ life. It is highly dependent on the plaque composition as it is more frequent in atheromas rich in macrophages and poor of fibroblasts and consequently in collagen fibers (29). However, plaque destabilization and rupture is still an unpredictable event and strategies to stabilize the lesion represent a challenging problem.

In the present review, we will illustrate the importance of purinergic signaling in modulating pro- and anti-atherogenic responses, particularly in T cells and macrophages. We will also highlight the potential of purinergic receptor agonists and antagonists for new therapeutic strategies to treat atherosclerosis.

## Pathogenesis of Atherosclerosis

Although abnormal lipid accumulation in the artery wall during atheroma formation is considered the main hallmark of the disease (30), there is still debate on triggering factors and stressors taking part in the initial stages of the disease. While there is a consensus on the participation of innate and adaptive immunity in chronic inflammation underlying atherogenesis, less is known on signals activating immune cells. Danger signals, i.e., pathogen-associated molecular patterns (PAMPs) derived from viruses and bacteria, as well as danger-associated molecular patterns (DAMPs), which are intracellular or endogenous molecules, have been linked to atherosclerosis (31). Among PAMPs, bacterial lipopolysaccharide (LPS), cytomegalovirus (CMV) and human immunodeficiency virus (HIV); while among DAMPs indicated to take part in atherogenesis: minimally modified LDL, oxidized LDL, oxidized phospholipids, advanced glycation end-products, high-mobility group box 1 and heat shock proteins. Interestingly, fatty acids can induce sterile vascular inflammation (**Figure 1**, topic 1) (32).

Cytokines play a fundamental role in atherosclerosis and associated comorbidities (e.g. psoriasis, SLE, CKD) (33–37). Pro- and anti-atherogenic cytokines have been shown. To the first group belong molecules with pro-inflammatory activities, such as interferons (IFNs) (α, β, γ), interleukin-(IL)1β, IL-6, IL-17a, granulocyte-macrophage colony-stimulating factor (GM-CSF), monocyte chemoattractant protein-1 (MCP-1), tumor necrosis factor (TNF)-α while among anti-atherogenic cytokines, transforming growth factor (TGF)-β, IL-10, and IL-35. The preeminent effect of pro-atherogenic cytokines ranges from induction of the synthesis of other cytokines, amplifying the pro-inflammatory activities of immune cells, to upregulation of endothelial adhesion molecules, thus favoring attachment and diapedesis of monocytes and lymphocytes (38).

## Purinergic Receptors

**Figure 2:**
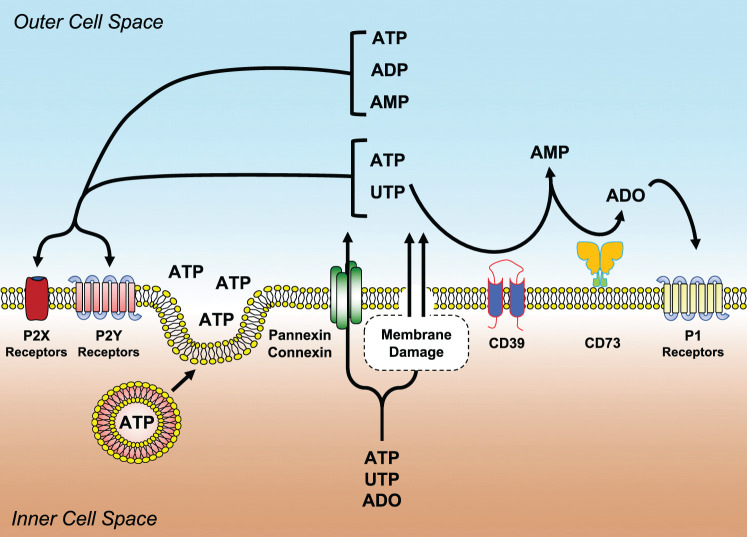
Main molecular components of the purinergic signaling network. Nucleotides (ATP, ADP, UTP, UDP, etc.) and nucleosides (ADO) can be released or transported extracellularly as a consequence of shear stress membrane damage, hypoxia, apoptosis, necrosis and infections. Once liberated, they bind and activate purinergic P2 (P2Y and P2X) or P1 (A_1_, A_2A_, A_2B_, A_3_) receptors. ADO is generated from the enzymatic conversion of ATP/ADP to AMP by the ectonucleoside triphosphate diphosphohydrolase CD39 and with the hydrolysis of AMP to ADO by the ecto-5′-nucleotidase CD73. ADO activates P1 receptors.

Nucleotides and nucleosides are not just accumulated and used within the cell but they are also secreted and synthesized extracellularly where they serve as intercellular messengers. ATP, ADP, UTP, UDP and adenosine, just to cite some, present at high concentrations within the cell where they exert multiple roles, bind extracellularly to evolutionary conserved P2 (activated by nucleotides) and P1 (activated by adenosine) plasma membrane receptors (**Figure 2**). Signal transduction of these receptors modulates cell and tissue pathways involved in tissue metabolism, gastrointestinal and hepatic function, circulation, nervous tissue response and immune defense (39–41). Interestingly, dysregulation of the purinergic signaling network has been implicated in the pathogenesis of allergic and neurological diseases, tissue fibrosis and cancer (42–46). Extracellular nucleotides participate in normal circulation physiology, but also in the onset of pathologic states that develop into the blood vessels, such as in blood hypercoagulability, thrombosis, atherosclerosis (40, 47, 48).

### P2 Receptors

#### P2X Receptors

They are grouped into two subfamilies, namely: P2X and P2Y (49) (**Figure 2**). P2X receptors comprise seven subtypes (P2X1-P2X7). They are highly conserved, trimeric, ATP-gated ion channels, selective for monovalent and divalent cations Na^+^, K^+^, Ca^2+^, Mg^2+^. Upon binding of the ligand, some of them desensitize (50, 51). Interestingly, the existence of lipid rafts and the level of cell membrane cholesterol can modulate the sensitivity of P2X receptors to ATP (52, 53). For the many responses they mediate in the circulatory apparatus, P2X receptors have been chosen as therapeutic targets for the cardiovascular system (54, 55).

The seventh subtype is an exception between P2X for its permeability transition and for not desensitizing in the presence of ATP (56). Its potential interest in atherogenesis is very high. Involvement of P2X7 in energy metabolism has been shown in mice; accordingly, deletion of the receptor induces lipid accumulation, fat mass distribution increase and gain of weight (57). The receptor is also endowed with the ability to induce transcription and secretion of inflammatory cytokines such as IL-1β, IL-18 (**Figure 1**, topic 5) and IL-6 which are central in atherosclerosis (58–60). Hence, Toll-like receptors (TLR) and P2 purinergic receptors induce activation of inflammasomes (61). Their activation by extracellular ATP causes IL-1β and IL-18 release (62). Interestingly, engulfment of lipids by macrophages increases the sensitivity of TLR to their ligands and activates NLRP3 (also known as NOD-, LRR- and pyrin domain-containing 3, NALP3) inflammasome (63, 64). NLRP3 is activated by two signals: the first being microorganisms or inflammatory cytokines endowed of the ability to activate transcription factor NF-κB, upregulate NLRP3 proteins and expression of the inactive form of the cytokines; while the second step is mediated by different stimuli among which extracellular ATP through activation of the P2X7 receptor (65). An important confirmation on the importance of P2X7 in atherogenesis comes from the animal model, where the absence of this subtype inhibits inflammasome activation and improves atherosclerosis (66).

#### P2Y Receptors

P2Y receptors include eight subtypes named: P2Y_1_, P2Y_2_, P2Y_4_, P2Y_6_, P2Y_11_, P2Y_12_, P2Y_13_ and P2Y_14_ (67). They have a membrane topology with seven-transmembrane domains and couple intracellularly to G_q_/G_11_ or G_i/0_ proteins (67) (**Figure 2**). They differ in agonist specificity. P2Y_1_, P2Y_12_ and P2Y_13_ subtypes are preferentially activated by ADP (68), whereas P2Y_6_ by UDP. P2Y_2_ is activated by UTP or ATP, while P2Y_4_ and P2Y_11_ are selective for UTP and ATP, respectively (69). P2Y_14_ is activated by UDP-glucose (69). P2Y receptors modulate several physiological responses.

Endotheliocytes release ATP in response to blood flow changes, hypoxia, or damaging agents (40, 70, 71). Moreover, ATP and other nucleotides are released from all dying cells and act as DAMPs activating and recruiting immune cells (58) (**Figures 1** and **2**). Interestingly, oxLDL favor nucleotide release from endothelial cells (72). Triggering of the P2Y_2_ receptor by ATP secreted by endothelial cells upon stimulation with oxLDL induces expression of receptors for advanced glycation end-products and adhesion molecules (73). Furthermore, the release of ROS and ATP/UDP from endothelial cells upon exposure to oxidized low-density lipoprotein (oxLDL), induce autocrine P2Y_1_-mediated upregulation of ICAM-1 and VCAM-1 with subsequent stimulation of leukocyte adhesion (74) (**Figure 1**, topic 2).

As an example, platelet aggregation is dependent on adenosine ADP/P2Y_12_-mediated amplification of thrombin effects. During platelet aggregation, the ADP receptor P2Y_12_ plays a pivotal procoagulant role as shown by the benefits gained by its inhibition with the receptor blocker Clopidogrel in patients with cardiovascular disease such as in acute coronary syndrome, recent stroke and arterial disease (75, 76). In abdominal aortic aneurysm, a condition characterized by dilatation of the abdominal aorta which involves antigen-driven T cells in the site of inflammation (77), Clopidogrel reduces the content of cytotoxic CD8^+^ T cells in the aortic wall and has an ameliorative role in the disease (78). Indeed, P2Y receptors would be central in inducing endothelium activation and atherogenic modifications at least in the double negative knockout (P2Y_1_^-/-^/ApoE^-/-^) mice. In these animals, the P2Y_1_ subtype contributes to TNF-α-induced ICAM-1 and VCAM-1 exposure with consequent leucocyte recruitment in inflamed femoral arteries (79). Moreover, reduction of the aortic sinus lesions associated to a decrease in macrophages infiltration and to a diminished VCAM-1 expression in endothelial cells of P2Y1^-/-^ApoE^-/-^ compared to ApoE^-/-^ mice suggests that atherosclerotic lesions are due to endothelial or smooth muscle cells expression of P2Y_1_ receptors (80). Interestingly, bacterial lipopolysaccharide (LPS) upregulates lectin-like oxLDL receptor in endothelial cells (81), which in turn induces a P2Y_1_- and P2Y_1_-mediated upregulation of ICAM-1 and VCAM-. This prompts leucocyte adhesion to endothelial cells (74). Similarly, the P2Y_2_ subtype promotes atherosclerosis in mice by inducing the expression of the same adhesion molecules. Matrix metalloproteinase-2 proteolytic activity was reduced in atheroma of P2Y_2_^-/-^ ApoE^-/-^ mice (82). Another potentially very interesting for the pathogenesis of atherosclerosis is the P2Y_6_ subtype. Hence, P2Y_6_ is upregulated during vascular inflammation induced by TNF-α or LPS stimulation in mice, and its inhibition or ablation reduces the vascular inflammatory response (83). These findings suggest that P2Y receptors could be targeted for therapeutic purposes in atherosclerosis.

### P1 Receptors

P1 receptors are activated by adenosine (ADO) that can be transported outside the cell by specific membrane transporters or generated extracellularly by ATP and ADP hydrolysis (see next paragraph) (**Figure 1**). ADO concentration in the extracellular fluids ranges from 100 to 500 nM in homeostatic conditions while it augments to low micromolar for the presence of inflammation or during hypoxia and ischemia (84, 85).

ADO or ADORA receptors consist of four subtypes: A_1_ (ADORA1), A_2A_ (ADORA2A), A_2B_ (ADORA2B) and A_3_ (ADORA3). They are seven-transmembrane G-protein-coupled receptors that associate with G-proteins. Depending on receptor subtype, ADO activates (A_2A_, A_2B_) or inhibits (A_1_, A_3_) adenylate cyclase (AC) (86). ADORA receptors also differ in ligand affinity, being A_1_, A_2A_ and A_3_ subtypes activated by low (10–50 nM) ADO concentrations while on the contrary, A_2B_ needs around 1 mM ADO for activation (87).

P1 receptors induce multiple responses (42, 85, 88). Extracellular ADO is very important to dampen acute inflammation thus preventing tissue injury. ADO-mediated immunosuppressive mechanisms are mainly based on inhibition of pro-inflammatory cytokine secretion, production of suppressive cytokines and induction of regulatory immune cells. Endothelial cells use adenosinergic signaling to regulate the leakiness through the endothelial monolayer of the brain capillaries, for the passive exchange of solutes and proteins (89, 90); however, the use of P1 agonists, particularly of the A_2A_ subtype has to be carefully evaluated for the side effects deriving from the T cell migration through the blood-brain barrier (91). Interestingly, A_2A_ receptor signaling has also been indicated as a target for limiting aneurysm formation (92); A_3_ antagonism reduces hypercholesterolemia in ApoE^-/-^ mice (93). Therefore, ADO and its receptors represent promising pharmacological targets to treat atherosclerosis.

### Ectonucleotidases

Extracellular nucleotide concentration in homeostatic conditions is low or close to zero. This is due to the hydrolyzing activity of different plasma membrane ectonucleotidases transforming ATP to ADP and then to ADO (**Figure 2**). Besides avoiding of accumulation of nucleotides in the extracellular *milieu*, these enzymes degrade P2 receptor agonists (i.e., nucleotides) lowering their concentration thus reducing the efficiency of stimulation. Conversely, their activity augments the amount of ADO thus increasing the probability of activating P1 receptors (42). Shifting from P2 to P1 activation has quite often the consequence of changing purinergic-mediated responses from pro- to anti-inflammatory, thus preserving tissue integrity (94).

Different ectonucleotidase families have been described: ectonucleotide pyrophosphatase/phosphodiesterase (NPP), alkaline phosphatases, ectonucleoside triphosphate diphosphohydrolases (NTPDases, among which CD39 or NTPDase1) and ecto-5’-nucleotidase (CD73). CD39 catalyzes the conversion of ATP or ADP to AMP, while CD73 hydrolyzes AMP to ADO (95–97) (**Figure 2**). Ectonucleotidases play a central role in immune regulation, thus preventing the development of conditions favoring autoimmune diseases (94). Moreover, the generation of ADO by ectonucleotidases reduces tissue damage and ameliorates tissue physiology in hypoxia-related disease states (98, 99). CD39 has been associated with resistance to thrombus formation in injured mice arteries (100) while in CD73^-/-^ mice, absence of the enzyme does not directly affect thrombosis, but indirectly lowers it by increasing CD39 expression, particularly on monocytes (101, 102). CD39 likely exerts multiple and sometimes apparently contrasting effects in atherosclerosis. The absence of this gene in hyperlipidemic mice decreases atheroma formation and it was hypothesized that this effect resulted from multiple contributions, i.e.: decreased platelet activation, increased plasma HDL concentration and augmented cholesterol efflux (103). Expression of CD39 is crucial in neointimal formation after vascular injury in mice as its absence impairs the migration of vascular smooth muscle cells (104).

## Modulation of Macrophages by Purinergic Signaling During Atherogenesis

### P2 Mediated Effects

Macrophages and DC express both P2X and P2Y receptor subtypes that are involved in modulating responses ranging from cytokine secretion, giant cell formation, production of oxygen radicals and antigen presentation.

Immunohistochemistry demonstrates that P2Y_6_ is upregulated in the atherosclerotic aortic segment of ApoE^-/-^ mice after 4-week of cholesterol-enriched diet, with the accumulation of P2Y_6_ expressing macrophages into the plaque. Interestingly, Suramin or PPADS treatments were able to reduce the plaque size, without modification of the number of macrophages and smooth muscle cells (105). P2Y_6_ receptor mRNA increases in aortic portions with atherosclerosis, while expression of the mRNA for other P2Y subtypes (P2Y1, P2Y_2,_ P2Y_4_) remain unchanged (105). However, the participation of the P2Y_6_ receptor to atherosclerosis in mice seems to be dependent on the experimental model used. A reduction in atherosclerotic plaque formation in the aortic arch was observed in high fat-fed LDLR knockout mice lacking the P2Y_6_ receptor in bone marrow-derived cells, but not in other mouse models (106). P2X7 is highly expressed in immune cells, particularly in macrophages where it is involved in IL-1β and IL-18 processing and release (59, 107–110). Macrophages are the main source of IL-1β, which is responsible for inflammation linked to atherosclerosis. It can thus be hypothesized that stimulation of P2X7 by extracellular ATP released within the atheroma induces the release of this pro-inflammatory cytokine (111–113). The efficacy of the A740003, a P2X7 specific antagonist, in decreasing vessel inflammation further supports its role in atherosclerosis and gives a new chance for the local pharmacological targeting of atherosclerosis (113).

IFN-γ is also a central mediator in atherosclerosis (114). IFN-γ potentiates IL-1β release from primary human monocyte-derived DC. Indeed, IFN-γ also upregulates expression of the P2X7 subtype, which in turn prompts IL-1β secretion (115, 116). IL-18 and its functional receptor have been detected in human endothelial cells, SMC and macrophages, and are implicated in atherogenesis (117). Since P2X7 expressed by human macrophages is also involved in ATP stimulated IL-18 release it again represents a suitable candidate for pharmacological targeting of atherosclerosis (108).

The centrality of NLRP3 inflammasome in atherosclerosis has also been well ascertained (118). Different approaches have been successfully attempted to inhibit the protein complex both *in vitro* and *in vivo*. This latter has shown a positive effect on experimentally induced atherosclerosis (119–121). Extracellular ATP is among stimuli that potently activate NLRP3, therefore, it is very promising for therapeutic purposes the observation that deficiency of a single purinergic receptor, namely the P2X7 subtype, is sufficient to block NLRP3 inflammasome and ameliorate the clinical picture of atherosclerosis in mice (122).

## Modulation of T Lymphocytes by Purinergic Signaling During Atherogenesis

### P2 Mediated Effects

P2Y and P2X receptor activation lead the inflammatory processes of the vessels favoring interactions between leukocytes, platelets and vessel wall. The P2Y_12_ subtype has attracted interest for its pro-thrombotic and pro-inflammatory role both in Apolipoprotein E-deficient mice and in humans (123). Contribution of the ADP receptor in modulating atherogenesis in the mouse model would be at least in part due to the induction of platelet α-granule release that would increase recruitment of inflammatory cells (124).

During atheroma formation, platelets induce a phenotype change and INF-*γ* secretion in human CD4^+^ T lymphocytes; but administration of the P2Y_12_ receptor blocker Prasugrel to human volunteers completely inhibits platelet-mediated pro-inflammatory changes induced in Th cells. Therefore, anticoagulant therapy with Prasugrel may provide therapeutic benefits both from direct platelet inhibition and also by downregulating the immune response (125). Clopidogrel, another P2Y_12_ inhibitor decreases expression of the purinergic receptor by leukocytes, ameliorates atheroma conditions and stabilizes aortic sinus plaques increasing the number of atheroprotective regulatory CD4^+^CD25^+^ T (Treg) cells in ApoE^-/-^ mice (126, 127). Although atherosclerosis is characterized by migration of different immune cells through the vessel wall, at least in the mouse model, lymphocytes are already present within the normal/noninflamed aorta before the onset of atheroma; while macrophages and DC that perform T cell antigen presentation are recruited into the artery wall. This migration is partially dependent on L-selectin (CD62L) both in normal and atherosclerosis-prone ApoE^−/−^ mouse aorta (128). Shedding of CD62L occurs during lymphocyte activation and rolling; interestingly, activation of the P2X7 receptor triggers the shedding of CD62L in leukocytes (**Figure 1**, topic 7) (129, 130).

### P1 Mediated Effects

It is long known that ADO has anti-inflammatory properties (58). Curiously, the potent anti-inflammatory drug methotrexate is responsible for ADO release that activating A_2_ receptors expressed by immune cells, reduces their presence in the inflamed tissue (131). Since ADO acts as a down-modulator of the immune response, it exerts atheroprotective functions by reducing the secretion of pro-inflammatory cytokines, thus lowering immune-mediated tissue damage (58). The role of CD8^+^ T lymphocytes in atherosclerosis has been the object of intense debate. However, a recent report has shed light on this issue and on the involvement of CD39 ectonucleotidase in conferring a regulatory and atheroprotective phenotype to CD8^+^ cells. This is associated with a reduction in cytokine production through increased CD39 expression in both mouse and human atherosclerotic lesions (132).

## Conclusions

Atherosclerosis is a leading cause of death in developed countries and it has been the target of multidisciplinary therapeutic approaches to reduce the relevant burden of life loss and health spending. Data coming from extensive epidemiological, clinical and experimental studies show that lifestyle habits are crucial to prevent atherosclerosis. Several strategies have been tested to treat the disease, among them: cholesterol-lowering agents, blood pressure reducing drugs, anti-inflammatory agents (corticosteroids, monoclonal antibodies to cytokines) and anti-P-selectin antibodies (133). Indeed, no definitive answers on the efficacy of these clinical approaches have been obtained. Therefore, novel therapeutic solutions are highly required (2, 33, 133).

ADO, for example, behaves as a down-modulator of immune cell activation as shown in many *in vitro* studies as well as in animal models and clinical trials. Besides anti-inflammatory properties and inhibition of cholesterol accumulation into the vessels, ADO also shows anti-thrombotic effects, thus having an atheroprotective potential sufficient to prompt clinical trials particularly involving the A2A receptor (134–137).

Macrophages and lymphocytes are central in the evolution of atherosclerosis for their ability to produce signals feeding the underlying pro-inflammatory background of the disease (19, 26, 38).

IL-1β has a pivotal role in atherosclerosis, and purinergic signaling is the main triggering way for its release. Interestingly, Losartan, an angiotensin II receptor blocker used to treat hypertension, inhibits LPS/ATP-induced IL-1β secretion by suppressing NLRP3 inflammasome (119). The NALP3/P2X7 tandem has a well-ascertained role in inflammation. An important result obtained in the animal model consists in the observation that the absence of the P2X7 subtype impairs lesional inflammasome activity and ameliorates the disease, pointing to the centrality of this receptor as a trigger of NLRP3 induced inflammation (122). Due to the importance of NLRP3 in atherosclerosis, different ways have been proposed for its inactivation (120, 121, 138). Interestingly, the P2X7 antagonist A740003 also shows an effect in decreasing IL-1β secretion and MMP9 activity in ex-vivo cultures of atheromatic cells, independently from NLRP3 (113). Therefore, further studies are needed to shed light on the activation of this latter P2X7 dependent proinflammatory pathway.

The P2Y_2_ receptor subtype has also been endowed with pro-inflammatory properties in the ApoE^-/-^ mouse model, and pro-thrombotic capacities in human coronary artery endothelial cells. It would therefore be worthy to pharmacologically target this receptor in the attempt of reducing inflammation and thrombosis in atherosclerosis (139). Involvement of the P2Y_6_ receptor in the inflammatory background underlying atherosclerosis has been shown both in mice and humans (105, 140). This subtype is expressed in murine atherosclerotic plaques and is involved in NO production and IL-6 secretion in murine macrophages (105). The P2Y6 receptor subtype plays a role in immune cell activation and recruitment to the arterial wall, most likely by inducing MCP-1 and CCR2 overexpression, accompanied by modulation of the CCL2-mediated signaling (106, 141, 142). Accordingly, leukocyte migration and lesion size induced by the P2Y_6_ agonist UDP are decreased in P2Y_6_R^−/−^ mice. Accordingly, mice deficient in both P2Y_6_ and low-density lipoprotein, LDL, receptor show lower atherosclerotic lesion sizes and lipid accumulation in the aorta. Recent studies on P2Y_6_ pro-inflammatory effects had shed light on vascular inflammation in the presence of bacterial LPS. The P2Y_6_ receptor antagonist MRS 2578 shows a positive effect in down modulating a nuclear factor κB reporter and expression of pro-inflammatory genes in human microvascular endothelial cells *in vitro* (83). Moreover, inflammation and uptake of cholesterol by macrophages are lower in atheroma of P2Y_6_^-/-^ mice, candidating the subtype as a therapeutic target for atherosclerosis (66, 106, 140, 143).

P2Y_12_ receptor represents a further very promising molecule for the treatment of the disease as its inhibitor Ticagrelor reduces cardiovascular events in patients with acute coronary syndrome and decreases inflammatory endothelial activation and vascular dysfunction in ApoE^-/-^ mice (144, 145). Moreover the efficacy of Prasugrel, another P2Y_12_ receptor blocker, in abolishing pro-thrombotic and pro-inflammatory responses of platelets and CD4^+^ T cells in humans, may also provide an indirect positive effect on the inflammatory response underlying the genesis of atheroma and also in cardiovascular diseases involving T cells (125). Concerning CD8^+^ T lymphocytes, although their identification in the atherosclerotic lesions has already been reported a few decades ago, however, both atheroprotective and pro-atherogenic roles have been proposed, depending on the animal or research model used (146). Different CD8^+^ subpopulations would have a particular role in atherosclerosis. Two putatively protecting phenotypes have been identified and would be MHC class I-restricted CD8^+^ lymphocytes and regulatory CD8+CD25+ T cells (146); moreover, a role has been attributed to CD39 ectonucleotidase in reducing IFN-γ and TNF-α production by CD8^+^ in atherosclerotic lesions in mice (132). Another important point is that inhibition of the A_2A_ receptor reduces the formation of foam cells, making this receptor putatively interesting to inhibit lipid accumulation within the intima (134, 135). Another issue to be further explored is the involvement of TLR receptors in atherosclerosis, being TLR9 a first candidate for future studies (138, 147). It would also be worthy to check whether the expression of TLR is modulated by nucleotides during atherosclerosis. Experiments performed in hypercholesterolemic mice showed that oxidized phospholipids are proatherogenic; therefore, it would be interesting to check whether extracellular ATP may amplify this response (148).

The attenuation of the inflammatory background of atherosclerosis would be a desirable first step to treat the disease; rapidly expanding knowledge on the effects mediated by extracellular nucleotides and nucleosides on immune and non-immune cells participating in atherosclerosis will hopefully give a new chance of introducing new therapeutic compounds to treat inflammation and therefore atherosclerosis (93, 149). Another challenge consists of finding new ways for the *in situ* delivery of anti-atherosclerotic drugs, to block atheroma progression and possibly revert it. Nano- and micro-particles could likely be a new and possibly efficient way to administer drugs directly to the atherosclerotic lesions (150).

## Author Contributions

DF, FC, and AL conceived the review and wrote the manuscript. FC prepared the figures. DM and CC checked and revised the manuscript. All authors contributed to the article and approved the submitted version.

## Funding

This manuscript was supported by local funds of the University of Ferrara (2019-FAR.L-CC_002, 2020-FAR.L-CF_003, 2019-FAR.L-MD_001, 2019-FAR.L-SP_001, 2020-FAR.L-SP_001).

## Conflict of Interest

The authors declare that the research was conducted in the absence of any commercial or financial relationships that could be construed as a potential conflict of interest.

## Abbreviations

AC, adenylate cyclase; ADO, adenosine; ADP, adenosine diphosphate; ATP, adenosine diphosphate; CKD, chronic kidney disease; DAMPs, damage-associated molecular patterns; DC, dendritic cells; GM-CFS, granulocyte-macrophage colony-stimulating factor; ICAM-1, intercellular adhesion molecule 1; IFN, interferon; IL, interleukin; LDL, low-density lipoproteins; LPS, lipopolysaccharides; MCP-1, monocyte chemoattractant protein-1; MMP9, Matrix Metallopeptidase 9; NLRP3, NACHT, LRR and PYD domains-containing protein 3; NO, nitric oxide; NTPDase, ectonucleoside triphosphate diphosphohydrolase; oxLDL, oxidized low-density lipoprotein; PAMPs, pathogen-associated molecular pattern; ROI, reactive oxygen intermediates; ROS, reactive oxygen species; SLE, systemic lupus erythematosus; SMC, smooth muscle cells; TGF-β, transforming growth factor β; TLR, toll-like receptors; TNF-α, tumor necrosis factor α; UDP, uridine diphosphate; UTP, uridine triphosphate; VCAM-1, vascular cell adhesion protein 1.
